# Type IIs restriction based combinatory modulation technique for metabolic pathway optimization

**DOI:** 10.1186/s12934-017-0659-z

**Published:** 2017-03-16

**Authors:** Lijun Ye, Ping He, Qingyan Li, Xueli Zhang, Changhao Bi

**Affiliations:** 1 Tianjin Institute of Industrial Biotechnology, Chinese Academy of Sciences, Tianjin, 300308 People’s Republic of China; 2 Key Laboratory of Systems Microbial Biotechnology, Chinese Academy of Sciences, Tianjin, 300308 People’s Republic of China; 3 School of Pharmacy, East China University of Science and Technology, Shanghai, 200237 People’s Republic of China

**Keywords:** Metabolic pathway optimization, Type IIs restriction, β-carotene, MVA pathway, Terpene

## Abstract

**Background:**

One of the most important research subjects of metabolic engineering is pursuing a balanced metabolic pathway, which is the basis of an efficient cell factory. In this work, we dedicated to develop a simple and efficient technique to modulate expression of multiple genes simultaneously, and select for the optimal regulation pattern.

**Results:**

A Type IIs restriction based combinatory modulation (TRCM) technique was designed and established in the research. With this technique, a plasmid library containing variably regulated *mvaE*, *mvaS*, *mvaK*_*1*_, *mvaD* and *mvaK*_*2*_ of the mevalonate (MVA) pathway were obtained and transformed into *E. coli* DXS37-IDI46 to obtain a β-carotene producer library. The ratio of successfully assembled plasmids was determined to be 35%, which was increased to 100% when color based pre-screening was applied. Representative strains were sequenced to contain diverse RBSs as designed to regulate expression of MVA pathway genes. A relatively balanced MVA pathway was achieved in *E. coli* cell factory to increase the β-carotene yield by two fold. Furthermore, the approximate regulation pattern of this optimal MVA pathway was illustrated.

**Conclusions:**

A TRCM technique for metabolic pathway optimization was designed and established in this research, which can be applied to various applications in terms of metabolic pathway regulation and optimization.

**Electronic supplementary material:**

The online version of this article (doi:10.1186/s12934-017-0659-z) contains supplementary material, which is available to authorized users.

## Background

As the development of Synthetic Biology and Metabolic Engineering, various microbial cell factories have been developed for producing value-added chemical compounds. However, engineering of cell metabolism often disturbs the metabolic network,triggers energetic and objective inefficiency inside the cell, and impedes cell metabolism [1]. Hence, one of the most important research subjects of metabolic engineering is pursuing the balanced metabolic network and pathways. Techniques have been developed to analyze metabolic pathways, including genome-scale models and C^13^-metabolic flux analysis. And there have been strategies developed to relieve the metabolic burden, such as enhancing respiration, co-utilizing nutrient resources, decoupling cell growth with production phases, and dynamic regulatory systems [1]. As for a specific metabolic pathway, gene expression level is the key effector of the pathway efficiency [2]. Lower expression of genes decreases metabolic pathway flux, while overexpressed genes may over-consume building blocks and cause cells metabolic burden [3]. Furthermore, imbalanced pathway may cause accumulation of pathway intermediates, some of which may even be toxic and jeopardize cell growth [4].

Due to the complexity of metabolic network in organisms and difficulty to precisely control expression of certain gene, it is almost impossible to rationally design and construct an optimized metabolic pathway. In most metabolic engineering projects, one common way was to modulate gene expression one by one [5]. With this strategy, the possibility to achieve an optimized regulation pattern is very low. A better solution was to analyze all possible expression levels of pathway genes in a combinatorial fashion. With this strategy, Yin et al. constructed a plasmid library containing the possible combinations of gene regulation patterns [6]. A similar strategy was employed by Xu et al. to optimize fatty acid pathway. Plasmids of various copy numbers were used to carry expression genes for the first round of optimization, which was followed by fine tuning expression with four RBS elements [7]. However, in their work, regulatory parts were limited and the plasmids were exhaustively constructed one by one, which limited the experiment outcome and made the process laborious. The same group also established a BioBrick based method with specially designed restriction adapters. Genes with regulatory elements could be iteratively integrated into the ePathBrick vectors to create a diversified expression library [8]. Similarly, Zelcbuch et al. created a plasmid library construction method to “span high-dimensional expression space” [9]. In their methods, the libraries were constructed with multiple rounds of plasmid construction, which made the practice very time consuming. In the work of Lee et al., a combinatorial library was established by Gibson assembly based method in one reaction [10, 11]. However, only five regulatory parts were employed, which decreased the diversity of the combinatorial library. Based on the extensive researches and great progress achieved by fellow researchers in this subject, we were able to develop a convenient method for constructing complex combinatorial expression library, which was aimed for optimizing a metabolic pathway with maximal outcome and minimal lab hours.

**Fig. 1:**
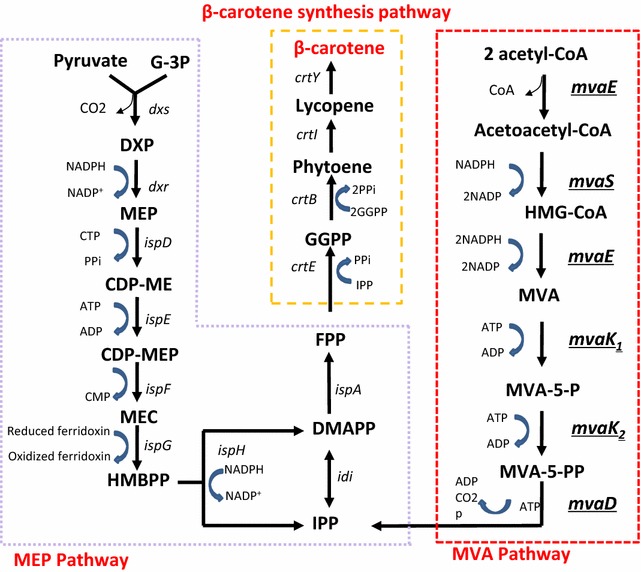
Illustration of β-carotene synthesis pathway in *E. coli* DXS37-IDI46 (pACYC184-AL-mva). This route involved β-carotene synthesis module, MEP module and MVA module. MVA Genes modulated with TRCM were underlined. *G*-*3*-*P* glyceraldehyde-3-phosphate, *DXP* 1-deoxy-d-xylulose-5-phosphate, *MEP* 2C-methyl-d-erythritol-4-phosphate, *CDP*-*ME* 4-diphospho-cytidyl-2*C*-methyl-d-erythritol, *CDP*-*MEP* 4-diphosphocytidyl-2C-methyl-d-erythritol-2-phosphate, *MEC* 2C-methyl-d-erythritol-2,4-cyclodiphosphate, *HMBPP* 1-hydroxy-2-methyl-2-(*E*)-butenyl-4-diphosphate, *HMG*-*CoA* 3-hydroxy-3-methyl-glutaryl-coenzyme A, *MVA* mevalonate, *MVA*-*5*-*P* mevalonate-5-phosphate, *MVA*-*5*-*PP* mevalonate-5-diphosphate, *IPP* isopentenyl diphosphate, *DMAPP* dimethylallyl diphosphate, *GPP* geranyl diphosphate, *FPP* farnesyl diphosphate, *GGPP* geranylgeranyl diphosphate

**Fig. 2:**
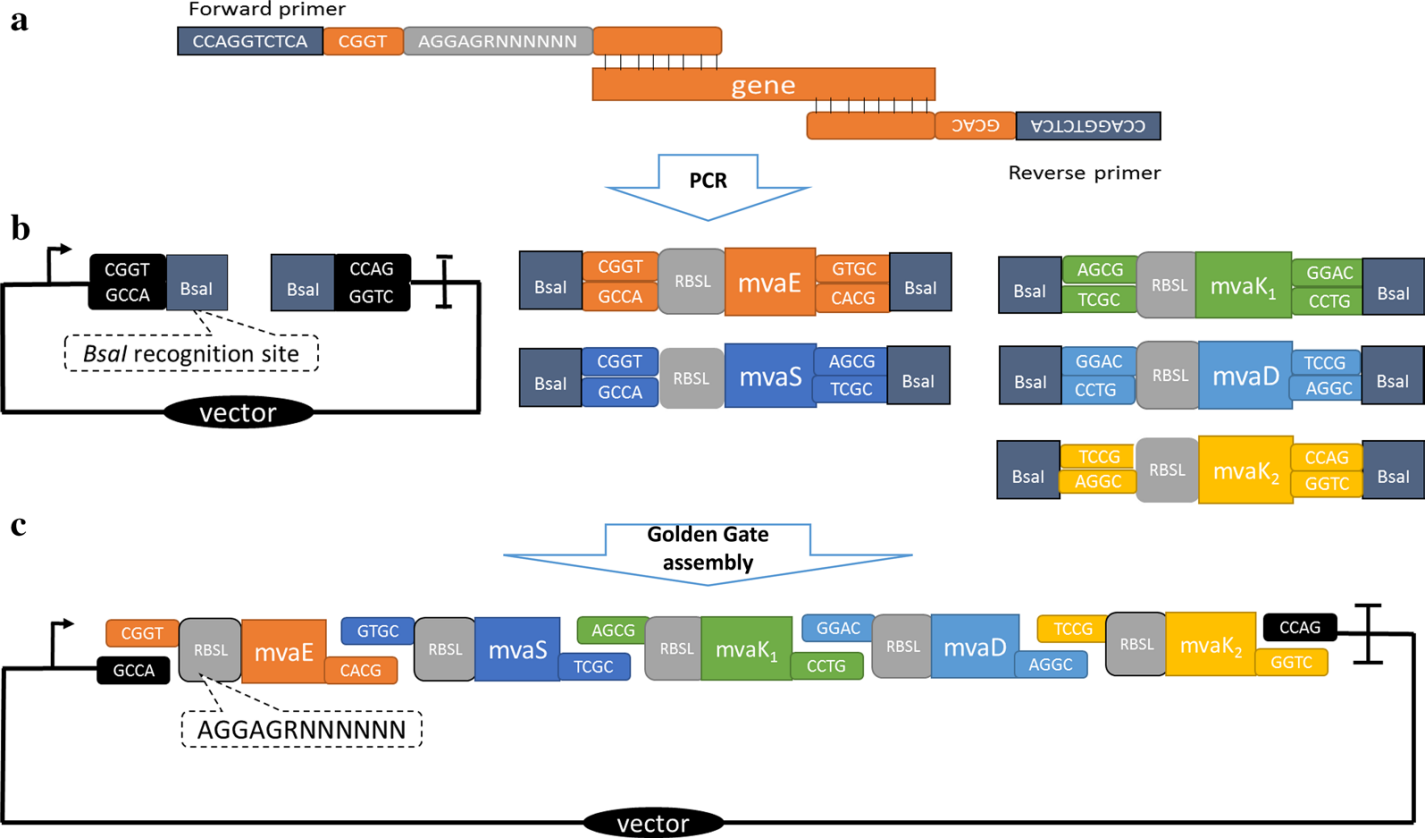
Modulation and optimization of MVA pathway with TRCM technique. **a** Primer design for obtaining assembly parts containing degenerate RBS sequences. Primers for amplification of MVA genes were embedded with *Bsa*I recognition site, optimized four bp linkers, and random RBS sequence AGGAGRNNNNNN. **b** TRCM DNA parts for assembly. One ready-made vector part was used to assemble with five library parts. **c** Combinatory expression library of MVA pathway. The plasmid library contained all five genes regulated with degenerate RBSs in various combinations

β-carotene, one kind of isoprenoids, is one of the strongest antioxidant in nature [12], and has tremendous potential in healthcare and pharmaceutical industries [13, 14]. Isoprenoids are all derived from two five-carbon building blocks, isopentenyl diphosphate (IPP) and dimethylallyl diphosphate (DMAPP), which are synthesized either by the mevalonate (MVA) pathway in eukaryotes, archaea, and some bacteria or 2-C-methyl-d-erythritol-4-phosphate (MEP) pathway in other prokaryotes and plastids in plants (Fig. 1) [13–15]. In MVA pathway, two acetyl-CoA are condensed into one atetoacetyl-CoA, which is then reduced into 3-hydroxy-3-methyl-glutaryl-coenzyme A (HMG-CoA). The CoA group is released from HMG-CoA to form MVA, which is phosphorylated into mevalonate-5-phosphate, and then mevalonate-5-diphosphate. This compound is coverted into IPP, the common precusor of isoprenoids (Fig. 1). The heterologous MVA pathways have been introduced into *E. coli* to improve precursor supply of IPP and DMAPP [16–20]. Isoprenoid production was improved by employment of the bottom portion of MVA pathway derived from *Streptococcus pneumoniae* and supplementation of MVA in culture [18, 20]. Lycopene production of *E. coli* with only native MEP pathway was increased twofold with introduction of the whole MVA pathway from *Streptomyces* sp. CL190 [19]. However, over-expression of mevalonate pathway genes was reported to inhibit cell growth. Pitera et al. found that accumulation of MVA pathway intermediate HMG-CoA inhibited cell growth, which was caused by overexpression of *atoB*, *mvaS* and *hmg1* [4]. Mevalonate kinase (MK), encoded by *erg12,* was identified as another rate-limiting enzyme when MVA pathway was adopted for amorphadiene production in *E. coli* [21]. Thus, the MVA pathway has to be expressed in an optimized and balanced status to benefit isoprenoid cell factories, otherwise an unbalanced MVA pathway would impede the growth and production. In this work, an MVA pathway optimized by combinatorial expression library technique was to be introduced into MEP pathway dependent *E. coli* cell factory for improving β-carotene production (Fig. 2).

## Methods

### Strains, medium and growth conditions

Strains used in this study are listed in Table 1. During strain construction, cultures were grown aerobically at 30 or 37 °C in Luria broth (per liter: 10 g Difco tryptone, 5 g Difco yeast extract and 5 g NaCl) and fermentation medium (per liter: 10 g Difco yeast extract, 15 g glycerol, 10.5 g K_2_HPO_4_.3H_2_O, 6 g (NH_4_)_2_HPO_4_, 5 g MgSO_4_·7H_2_O, 1.84 g citric acid monohydrate and 10 mL microelements solution; Microelements solution per liter: 10 g FeSO_4_·7H_2_O, 5.25 g ZnSO_4_·7H_2_O, 3.0 g CuSO_4_·5H_2_O, 0.5 g MnSO_4_·4H_2_O, 0.23 g Na_2_B_4_O_7_·10H_2_O, 2.0 g CaCl_2_ and 0.1 g (NH_4_)_6_Mo_7_O_24_). For β-carotene production, single colonies were picked from LB plate and inoculated into 15 × 100 mm tubes containing 4 mL LB with or without 34 mg/L chloramphenicol, and grown at 30 °C and 250 rpm overnight. Seed culture was subsequently inoculated into 100 ml flask containing 10 mL fermentation medium at an initial OD_600_ of 0.05, with or without 34 mg/L chloramphenicol, and grown at 30 °C and 250 rpm. After growth for 24 h, cells were collected for measurement of β-carotene production.

**Table 1:** Strains and plasmids in this study

Strains/plasmids	Relevant characteristics	Source/notes
Strains		
*E. coli* DH5α	F^−^ *endA1thi*-*1 recA1 relA1 gyrA96deoRΦ*80*dlac*Δ(*lac*Z) M15 Δ (*lacZYA*-*argF*) *U169hsdR17* (r_K_^−^, m_K_^+^) λ^−^ *supE44 phoA*	Invitrogen
CGMCC 1.2135	*Enterococcus faecalis* wild-type	CGMCC
CGMCC 1.8722	*Streptococcus Pneumoniae* wild-type	CGMCC
Dxs37-Idi46	ATCC 8739, *ldhA::*M1-12*::crtEXYIB::ldhA*, M1-37::*dxs*, M1-46::*idi*	Laboratory stock
Plasmids		
pACYC184-PgadA-RFP	*E. coli* expression vector derived from pACYC184, promoter of *gadA*, RFP, cat	Laboratory stock
pACYC184-AL-mva	plasmid library of combinatorically regulated MVA pathway, derived from pACYC184-PgadA-RFP	This study

### Genes, vector and primers

MVA pathway genes *mvaE, mvaS, mvaK*_*1*_, *mvaD,* and *mvaK*_*2*_ were amplified from genomic DNA of *Enterococcus faecalis* CGMCC No.1.2135 using primer set Ga2-R1-EfmvaE-F/Ga2-R1-EfmvaE-R, Ga3-R1-EfmvaS-F/Ga3-R1-EfmvaS-R, and from genomic DNA of *Streptococcus pneumoniae* CGMCC No.1.8722 using primer set Ga46-R1-SpmvaK1-F/Ga46-R1-SpmvaK1-R, Ga7-R1-SpmvaD-F/Ga7-R1-SpmvaD-R, and Ga8-R1-SpmvaK2-F/Ga8-R1-SpmvaK2-R respectively (Additional file 1: Table S1). The DNA fragments used for assembly were gel purified and designated as Ga2-mvaE, Ga3-mvaS, Ga46-mvaK1, Ga7-mvaD and Ga8-mvaK2 (Fig. 1b). Vector Fragment Ga91-184A was amplified from pACYC184-PgadA-RFP, and subjected to *Dpn*I digestion (10 U, 16 h, 37 °C) and gel purification. PCR was performed with PrimeSTAR^®^ HS DNA Polymerase (Takara) with primers purchased from GENEWIZ (Suzhou, China). All assembly primers were designed with optimized linkers for Type IIs restriction enzyme based assembly, in which forward primers for amplification of genes were embedded with an RBS library at 5′ ends. Primers used in this study are summarized in Additional file 1: Table S1.

### Construction of *mva* operon variants using TRCM

DNA fragments were assembled by Golden Gate DNA assembly method [22, 23]. 100 nanogram vector fragment Ga91-184A and equimolar amount of PCR amplified genes Ga2-mvaE, Ga3-mvaS, Ga46-mvaK1, Ga7-mvaD and Ga8-mvaK2 were mixed in 20 μL Golden Gate reaction solution with 1 μL* Bsa*I-HF, 1 μL T4 ligase (New England Biolabs, Ipswich, MA) and 1× T4 ligase buffer. The reaction was carried out in a thermocycler using the following program: 37 °C for 5 min, 37 °C for 5 min (step 2), 16 °C for 10 min (step 3), step 2 and 3 for 20 cycles, 16 °C for 20 min, 37 °C for 30 min, 75 °C for 6 min, and 4 °C for hold. After PCR, 0.5 μL plasmid-safe nuclease (Epicenter), and 1 μL of 25 mM ATP was added to the reaction, which was incubated at 37 °C for 15 min. 1.5 μL of the resultant reaction solution were transformed into 80 μL competent DXS37-IDI46 cells to obtain the library [24].

In order to determine whether the MVA pathway genes were successfully incorporated into vector backbone, recombinant clones were subjected to colony PCR analysis. PCR primers were designed to amplify the region from *mvaE* (the first gene on plasmid) to *mvaK*_*2*_ (the last gene on plasmid) by primers fE-JF and pK2-JR (Additional file 1: Table S1), which had a product size of 3.8 Kbp. A master mix with 1× Es Taq MasterMix (CWBio, Peking, China) and 0.4 μM forward and reverse primer (Additional file 1: Table S1) was prepared, and 20 μL master mix was dispensed into each PCR tubes. Colonies were directly transferred from LB agar plates into the PCR tubes with sterile toothpicks. The PCR cycling was started with an initial denaturation temperature at 94 °C for 10 min, followed by 30 cycles (94 °C, 30 s; 61 °C, 30 s; and 72 °C, 2 min) and one fill-up cycle (72 °C, 2 min). The PCR products were analyzed on agarose–TAE gels.

### Measurement of β-carotene titer

Production of β-carotene was quantified by measuring absorption of acetone-extracted β-carotene at 453 nm as previously reported [24]. A standard curve was obtained by measuring OD_453_ of β-carotene standard samples (Cat. No. C4582, Sigma, USA) with varied concentrations using a Shimadzu UV-2550 spectrophotometer (Shimadzu, Kyoto, Japan). The results represented the mean ± standard deviation (S.D.) of three independent experiments. Dry cell weight (DCW) was calculated based on optical density at 600 nm (1 OD_600_ = 323 mg DCW).

### Calculation of MVA pathway genes RBS strength of strains from TRCM libraries

RBS sequences of *mvaE, mvaS*, *mvaK*_*1*_, *mvaD,* and *mvaK*_*2*_ in representative strains were obtained by PCR and DNA sequencing. Their theoretical RBS strength characterized by the value of translation initiation rate was calculated with the RBS Calculator [25, 26]. The RBS sequence diversity of the combinatory library was analyzed with the Weblogo software [27].

## Results and discussions

### Design of a Type IIs restriction based combinatory modulation technique (TRCM) for metabolic pathway optimization

With the purpose of developing a simple technique to modulate and optimize expression of multiple genes simultaneously, we designed a Type IIs restriction based combinatory modulation technique (TRCM) for metabolic pathway optimization as illustrated in Fig. 2. Variably regulated genes were obtained by PCR amplification with extended primers, in which degenerate RBS nucleotides were embedded at the 5′ ends. Specifically designed linkers for Type IIs restriction enzymes were also imbedded in the primers to ensure the assembly pattern and efficiency.

Type IIs restriction based Golden Gate [23] was employed as DNA assembly method in this work, which has several advantages. First, there is no PCR reaction involved in the assembling process, which reduces the possibility of mutation compared with other PCR based assembly methods such as Gibson and CPEC [10]; second, the ligase facilitated irreversible ligation greatly improves assembly efficiency compared with homologous arm based method [23]. With this method, gene parts of a pathway were assembled with a vector part to form an expression plasmid. Since each gene part was constructed to carry a collection of regulatory parts, a combinatory plasmid library with variably regulated pathway genes was created, which was subsequently transformed into dedicated host to be screened and selected for strains carrying optimized pathways.

This technique was designed with the modularized strategy to be as simple as possible. The vector part was ready-made for all reactions, providing a stable plasmid backbone. By incorporation of fixed linkers and regulatory elements in primers for amplification of genes, the only variable parts of this method were the actual PCR primer sequences of pathway genes (Fig. 2a).

### Development and application of TRCM for MVA pathway optimization

Our lab has constructed a few *E. coli* β-carotene producers, such as DXS37-IDI46 and CAR001, by modulating several key genes of the MEP pathway module, the pentose phosphate pathway (PPP) module, the ATP module and the tricarboxylic acid cycle (TCA) module [24]. In this work, a heterologous MVA pathway optimized with TRCM technique was introduced into DXS37-IDI46 for further improving its β-carotene production (Fig. 2).

Since MVA pathway upstream genes *mvaS, mvaE* from *Enterococcus faecalis* and downstream genes *mvaK*_*1*_, *mvaK*_*2*_, *mvaD* from *Streptococcus pneumoniae* were reported to be successfully expressed in *E. coli*, they were selected to be used in this research [17]. As designed with the TRCM technique, primers for PCR amplification of vector and MVA genes were embedded with *Bsa*I recognition sites GGTCTC and specific four bp linkers, in order to be assembled in sequence. These linkers were rationally designed and experimentally tested to enable efficient assembly of DNA parts regardless of condition change (Fig. 2a).

To create a library of differently regulated genes, the RBS sequences of each gene were degenerated. For this purpose, forward primers of MVA genes Ga2-R1-EfmvaE-F, Ga3-R1-EfmvaS-F, Ga46-R1-SpmvaK1-F, Ga7-R1-SpmvaD-F and Ga8-R1-SpmvaK2-F were embedded with the random RBS sequence AGGAGRNNNNNN behind the 4 bp linkers. The starting code ATG of each gene was located behind the six Ns, which was also the starting point of actual PCR primers (Fig. 1a).

Gene parts, which carried front and back linkers for assembly in sequence, were obtained with PCR amplification. In Golden Gate assembly reaction, PCR amplified *mvaS, mvaE, mvaK*_*1*_, *mvaK*_*2*_ and *mvaD* parts were mixed with the ready-made vector part Ga91–184A (Fig. 1b). After reaction, a plasmid library was created, which had differentially regulated MVA genes in various combinations. Theoretically, all patterns of differently expressed MVA pathway could be obtained in such a library (Fig. 1c). With this simple method, we achieved the goal of spanning high-dimensional expression space [9].

### β-carotene production was improved with TRCM optimized MVA pathway

**Fig. 3:**
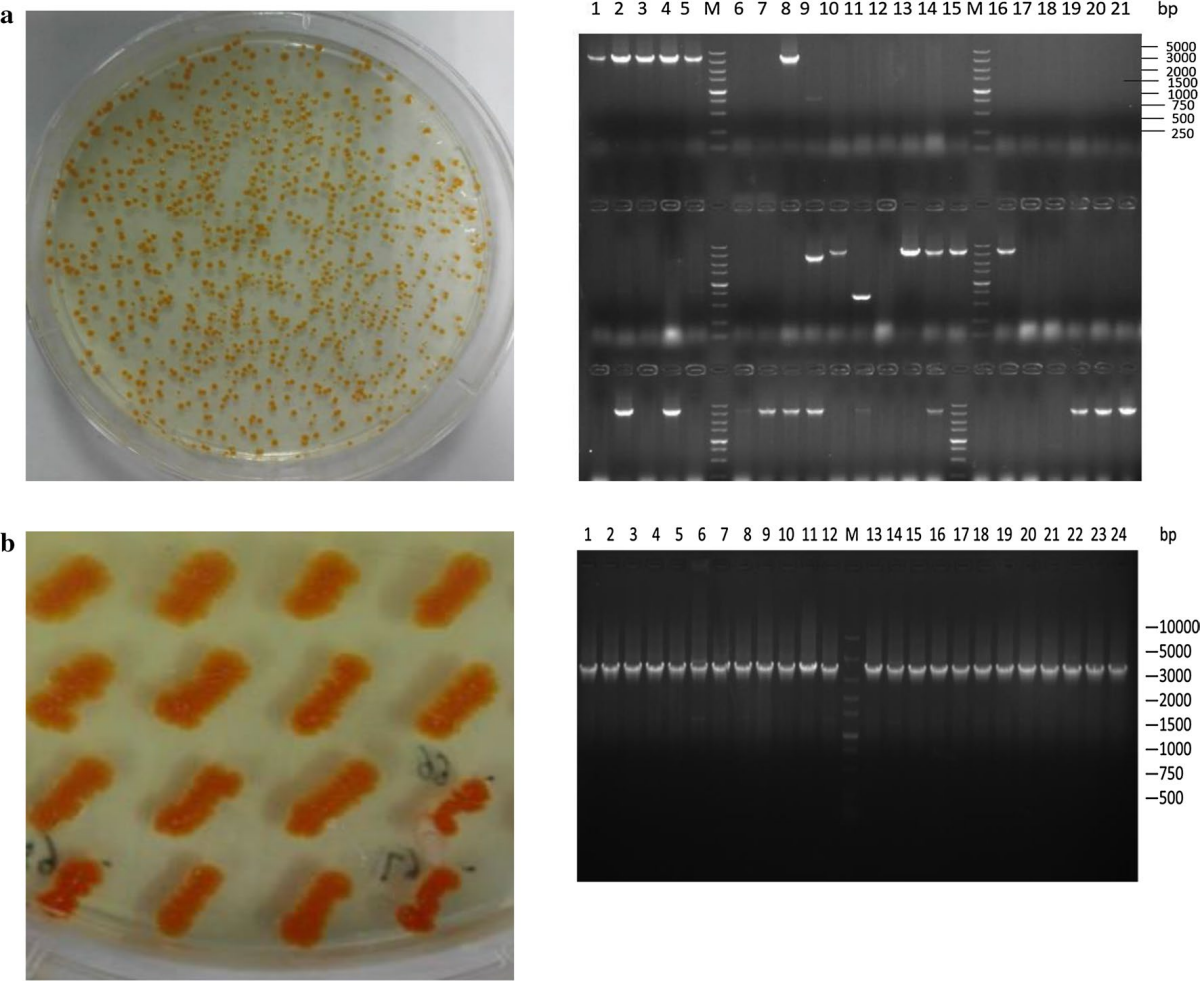
Determination of successfully assembled combinatory expression library pACYC184-AL-mva in host DXS37-IDI46. A 35% successful assembly ratio was determined by PCR analysis of randomly selected colonies, and a 100% assembly ratio was achieved with a color based pre-screening process. **a** DXS37-IDI46 transformed with TRCM assembly reaction solution after overnight incubation on LB plate. Colonies were randomly picked and analyzed by colony PCR to determine ratio of successfully assembled pACYC184-AL-mva. *M* marker, *1*–*21* colonies picked from LB plate. **b** Colonies from original plates were picked and evenly re-streaked on fresh chloramphenicol LB plates, then were validated by colony PCR. *M* marker, *1*–*24* colonies picked from the plate

To select for the optimally expressed MVA pathways, Golden Gate reaction solution containing plasmid library was transformed into the β-carotene producer strain DXS37–IDI46 (Table 1). After electroporation, transformed cells were plated on solid LB with 34 mg/L chloramphenicol to select for plasmid bearing transformants. Orange colored colonies with various intensity appeared on the plates as expected after incubation overnight (Fig. 3a), which indicated their different capacity of β-carotene production. Colonies were randomly picked and analyzed by colony PCR to determine the ratio of successful assembly, for which PCR primers were designed to amplify the region from *mvaE* to *mvaK*_*2*_ with primers fE-JF and pK2-JR (Additional file 1: Table S1), with an expected product size of 3.8 Kbp. As illustrated in Fig. 3a of TAE gel electrophoresis, 22 of 63 screened colonies had PCR bands of the correct size, which indicated a 35% successful assembly ratio of the synthetic operon. To determine this ratio of strains with increased β-carotene production, transformants were picked and evenly re-streaked on fresh chloramphenicol LB plates, which was to improve the reliability of color based β-carotene production screening (Fig. 3b). Colonies with deeper orange color were selected for PCR analysis. The gel electrophoresis (Fig. 3b) indicated a positive ratio of 100%, since all 24 strains gave the correct PCR products. The results indicated a decent assembly ratio of 35% was achieved by TRCM technique with modulation of five genes simultaneously, and a higher successful ratio could be achieved with a simple color based pre-screening process.

**Fig. 4:**
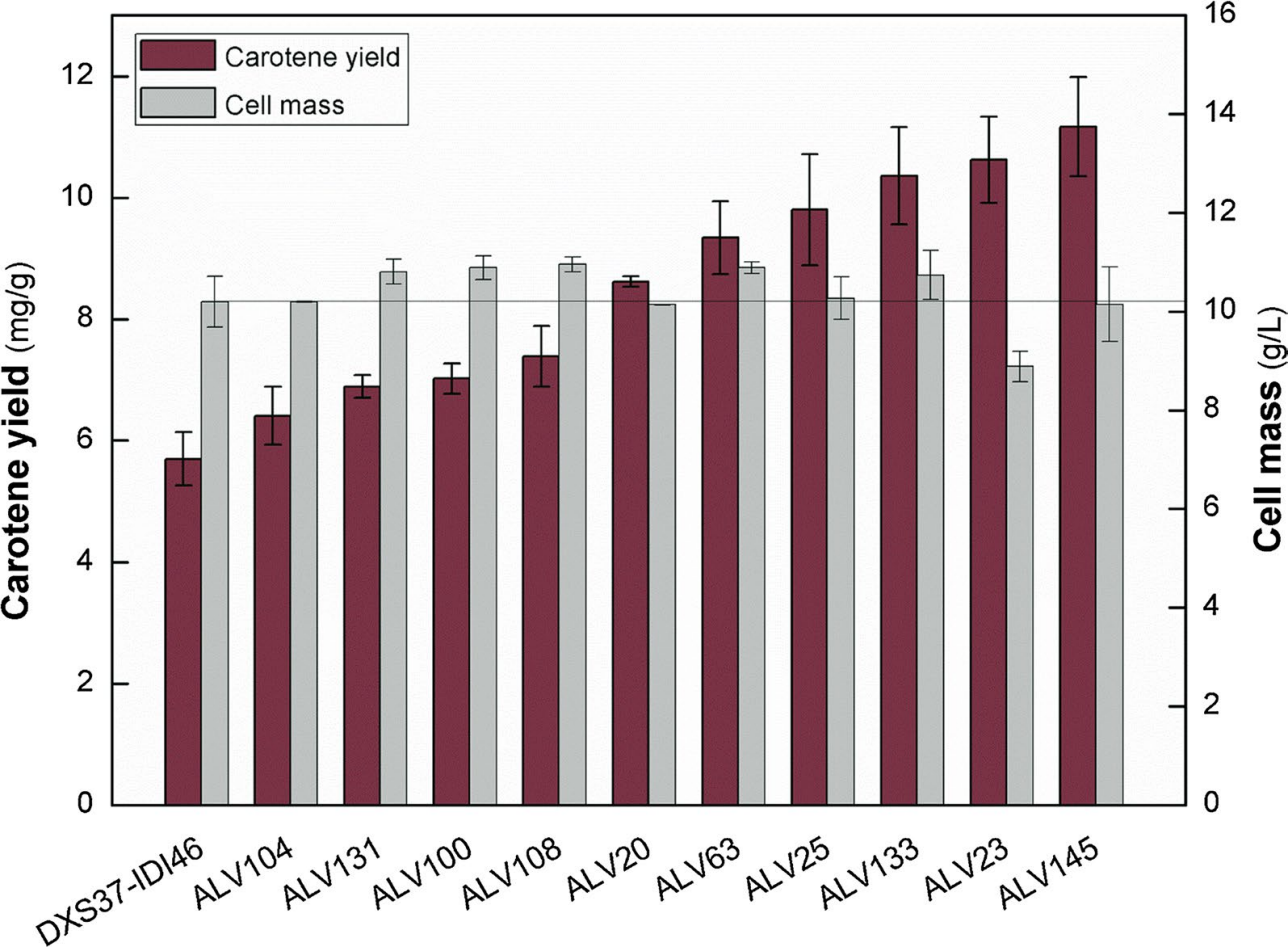
β-carotene yield and cell mass of representative strains from the combinatory expression library DXS37-IDI46 (pACYC184-AL-mva). Strains include ALV104, ALV131, ALV100, ALV108, ALV20, ALV63, ALV25, ALV133, ALV23, ALV145 and parent strain DXS37-IDI46. Three repeats were performed for each strain, and the *error bars* represented standard deviation

To select strains with significantly improved β-carotene producing capacity, colonies with deeper color were cultured aerobically, and the β-carotene titer was measured. Ten representative strains ALV104, ALV131, ALV100, ALV108, ALV20, ALV63, ALV25, ALV133, ALV23, and ALV145 were determined to have improved β-carotene production to various extent in comparison with DXS37-IDI46, as illustrated in Fig. 4. Strain ALV145 had the highest yield of 11.17 ± 0.82 mg/g, which was a 96.0% increase compared with the parent strain DXS37-IDI46. The significant improvement indicated that an efficient cell factory with optimized metabolic pathway could be obtained by the simple TRCM technique.

### A combinatorial expression library with five genes regulated by diverse RBSs was obtained with TRCM technique

To analyze the degenerated RBS sequences regulating MVA gene, PCR was used to amplify regions containing the RBS sequences of MVA operon genes in representative strains. The PCR products were sequenced subsequently to obtain the RBS sequence information, which was summarized in Table 2. As designed, a combinatorial expression library with the five MVA genes regulated by diverse RBSs was obtained. For each strain, RBSs of the five genes were all different; and for each gene, all ten RBSs were not same either. Diversity of the degenerate RBS sequence RNNNNNN from combinatory library was further analyzed with the Weblogo software [27]. A good but not great diversity was obtained for each of the five MVA genes, probably due to the low sample number, which was only ten for one gene. However, when all fifty RBS sequences were subjected to calculation, a logo with very high diversity was achieved (Additional file 2: Figure S1). In addition, among the fifty sequenced RBSs, ratio of the highest calculated RBS strength to the lowest was 183,455/92 [25, 26], which indicated an expression dynamic range of about 2000-fold. The results proved that TRCM technique process did not create significant bias, and were able to generate quite diverse combinatory expression library.

**Table 2:** MVA gene RBS sequences with their calculated strength of representative strains from the combinatory expression library DXS37-IDI46 (pACYC184-AL-mva)

Strains	The genes RBS of MVA pathway										Relative yield (%)^b^
	*mvaE*		*mvaS*		*mvaK*_*1*_		*mvaK*_*2*_		*mvaD*		
	Sequence	Calculated strength^a^	Sequence	Calculated strength	Sequence	calculated strength	Sequence	Calculated strength	Sequence	Calculated strength	
ALV104	AGGAGGCTAGAA	17988	AGGAGAGTTTTA	16327	AGGAGAGTCTTT	12463	AGGAGGGGGTGC	30731	AGGAGATTCCTG	92	112.5
ALV131	AGGAGGCTTTGG	8370	AGGAGGTGGGGG	162464	AGGAGGTATTTC	183455	AGGAGGTTGCGT	90504	AGGAGAATGGGC	612	120.9
ALV100	AGGAGGGTTTGA	9158	AGGAGGGTGGGG	72267	AGGAGATTGTGC	7513	AGGAGGGTGTCC	29379	AGGAGAATGGGG	447	123.2
ALV108	AGGAGGTTGGTG	8110	AGGAGAAATTGG	2064	AGGAGGGGGGGT	35173	AGGAGGGGGGTT	15646	AGGAGAGTTTCG	259	129.6
ALV20	AGGAGGGGGCTA	15575	AGGAGACTTTGT	7615	AGGAGGGCGCCT	6080	AGGAGGTTTGCT	103587	AGGAGGTATGCT	3238	151.2
ALV63	AGGAGGAGGTGT	33777	AGGAGGCATGGG	44050	AGGAGAAACGGC	30183	AGGAGGGGTCTA	14690	AGGAGGTTGCAT	5813	163.9
ALV25	AGGAGACACAAT	10817	AGGAGGGCGGTT	15646	AGGAGGGCTTTT	25668	AGGAGGTGC	1258	AGGAGGCTTCGT	559	171.9
ALV133	AGGAGAGTTTAC	25607	AGGAGGACGAGG	17119	AGGAGGGCTCCT	22126	AGGAGGGCGGGC	12493	AGGAGATCGGCC	92	181.8
ALV23	AGGAGGGAGTAA	50645	AGGAGGTGGGTG	148479	AGGAGAGGGTCC	8332	AGGAGGGATGCG	11418	AGGAGGTGCCAT	1870	186.3
ALV145	AGGAGGGATTGT	33777	AGGAGGAGCTCT	57705	AGGAGGTGGTGT	160285	AGGAGGGGGTGC	30731	AGGAGGTCTTGC	1258	196.0

^a^Calculated Strength was represented by the translation initiation rate calculated by RBS Library Calculator [9, 17]

^b^Relative yield compared with their parent strain DXS37-IDI46

### An approximate expression pattern of optimal MVA pathway was illustrated

**Fig. 5:**
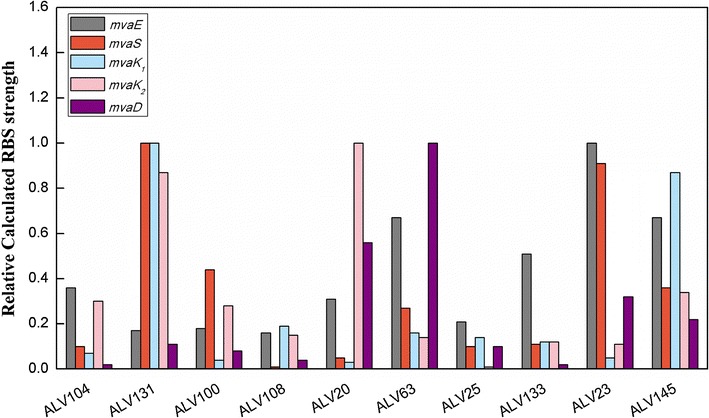
The relative calculated RBS strength of MVA genes in representative strains from library DXS37-IDI46 (pACYC184-AL-mva). The highest RBS strength for each gene was defined as 1, and a relative RBS strength of the ten strains for this gene was calculated accordingly. The ten strains are lined up along the *X axis* according to their β-carotene yield from low to high

To illustrate a general and approximate expression pattern of an efficient MVA pathway in *E. coli* cell factories, RBS strength of the MVA genes was analyzed with the RBS Calculator [25, 26]. Calculated RBS strength was represented by the translation initiation rate as listed in Table 2. The calculated RBS strength was by no means a very accurate measurement of the expression status of *mvaE*, *mvaS*, *mvaK*_*1*_, *mvaD* and *mvaK*_*2*_, however, could give a good estimation of a general trend of the optimized expression status of MVA pathway. For better illustration, the highest RBS strength for each gene was defined as 1, and a relative RBS strength of the ten strains for this gene was calculated accordingly (Fig. 5). The ten strains are lined up along the X axis according to their β-carotene yield. MVA gene expression pattern of ALV145 strain with the highest yield was obvious, that all genes were regulated to a medium level. In contrast, some of the inefficient strains had one or more MVA genes fell to very low expression level, or one or more genes reached the highest level. It was reported that some MVA pathway intermediates were toxic, for example HMG-CoA, accumulation of which affected cell growth and pathway efficiency [4]. Besides, Mevalonate kinase (MK), encoded by *mvaK*_*1*_, was identified as a rate-limiting enzyme [21]. Thus in an optimized MVA pathway, *mvaE* should be coordinately expressed with *mvaS* to avoid HMG-CoA accumulation, and a higher expression of *mvaK*_*1*_ is desired. It was found by the expression analysis, except ALV131, most of the inefficient strains did not follow these two rules.

Analysis of the representative strains indicated that an efficient MVA pathway contained genes expressed at a medium level, among which *mvaE* coordinately expresses with *mvaS* to avoid HMG-CoA accumulation, and a higher expression level of *mvaK*_*1*_ is beneficial.

## Conclusion

A TRCM technique was designed and established in the research, which could be easily applied to various applications in terms of metabolic pathway regulation and optimization. An optimized MVA pathway was constructed with TRCM to increase β-carotene yield of *E. coli* cell factory by twofold, and the optimal regulation pattern of MVA pathway was analyzed and illustrated.

## Additional files

**Additional file 1: Table S1.** Oligonucleotides used in this study.

**Additional file 2: Figure S1.** The sequence logo diagrams of the degenerate nucleotides in RBSs. Plasmid maps and DNA sequences.

## Abbreviations

LB: lysogeny broth
RBS: ribosome-binding site
DCW: dry cell weight
ATP: adenosine triphosphate
