# Supplementary material for: Type IIs restriction based combinatory modulation technique for metabolic pathway optimization
Source: Microb Cell Fact. 2017 Mar 16;16:47. doi: 10.1186/s12934-017-0659-z (PMC5353881; doi:10.1186/s12934-017-0659-z)
Supplement: Supplementary file 1 — Additional file 1: Table S1. Oligonucleotides used in this study. [file 12934_2017_659_MOESM1_ESM.docx]

**Table S1** Oligonucleotides used in this study.

| Primers | Sequences | Source |
| --- | --- | --- |
| Ga2-R1-EfmvaE-F | CCAGGTCTCACGGTAGGAGRNNNNNNATGAAAACAGTAGTTATTA TTGAT | This study |
| Ga2-R1-EfmvaE-R | CCAGGTCTCAGCACTTATTGTTTTCTTAAATCATTTAAAATAG | This study |
| Ga3-R1-EfmvaS-F | CCAGGTCTCAGTGCAGGAGRNNNNNNATGACAATTGGGATTGAT AAAATTAGT | This study |
| Ga3-R1-EfmvaS-R | CCAGGTCTCACGCTTTAGTTTCGATAAGAGCGAACGGTATTATTA | This study |
| Ga46-R1-SpmvaK1-F | CCAGGTCTCAAGCGAGGAGRNNNNNNATGACAAAAAAAGTTGG TGTCGGTCAGG | This study |
| Ga46-R1-SpmvaK1-R | CCAGGTCTCAGTCCTTACAGGCTCTCTATCCATGTCTGAACAG | This study |
| Ga7-R1-SpmvaD-F | CCAGGTCTCAGGACAGGAGRNNNNNNATGGATAGAGAGCCTGTA ACAGTA | This study |
| Ga7-R1-SpmvaD-R | CCAGGTCTCACGGATTAACAGCAATCATCTTGACTCA | This study |
| Ga8-R1-SpmvaK2-F | CCAGGTCTCATCCGAGGAGRNNNNNNATGATTGCTGTTAAAACTT GCGGAA | This study |
| Ga8-R1-SpmvaK2-R | CCAGGTCTCACTGGTTACGATTTGTCGTCATGTCCTAT | This study |
| Ga9-184-F | CCAGGTCTCACCAGGTGCTTAAGGGATCCAAAC | This study |
| Ga1-gadA-R | CCAGGTCTCAACCGTAAATTTATTTGAAGGCAATAAAAAAGTAG | This study |
| fE-JF | CAGTTCCGCTTGCGTTAGCC | This study |
| pK2-JR | ACGACGCTGCCACTAGAACC | This study |
