# Supplementary material for: Type IIs restriction based combinatory modulation technique for metabolic pathway optimization
Source: Microb Cell Fact. 2017 Mar 16;16:47. doi: 10.1186/s12934-017-0659-z (PMC5353881; doi:10.1186/s12934-017-0659-z)
Supplement: Supplementary file 2 — Additional file 2: Figure S1. The sequence logo diagrams of the degenerate nucleotides in RBSs. Plasmid maps and DNA sequences. [file 12934_2017_659_MOESM2_ESM.docx]

**Supplementary plasmid profiles and gene sequences**

pACYC184-PgadA-RFP


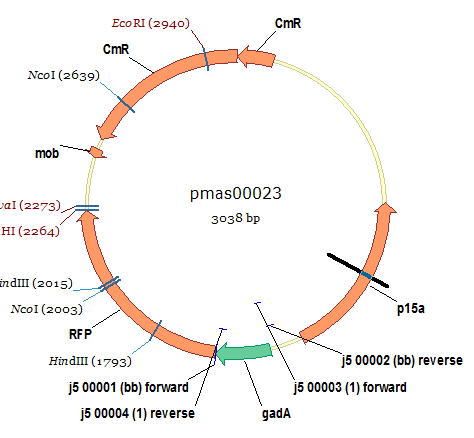


>pACYC184-gadA-RFP

TCCAGCTGAACGGTCTGGTTATAGGTACATTGAGCAACTGACTGAAATGCCTCAAAATGTTCTTTACGATGCCATTGGGATATATCAACGGTGGTATATCCAGTGATTTTTTTCTCCATTTTAGCTTCCTTAGCTCCTGAAAATCTCGATAACTCAAAAAATACGCCCGGTAGTGATCTTATTTCATTATGGTGAAAGTTGGAACCTCTTACGTGCCGATCAACGTCTCATTTTCGCCAAAAGTTGGCCCAGGGCTTCCCGGTATCAACAGGGACACCAGGATTTATTTATTCTGCGAAGTGATCTTCCGTCACAGGTATTTATTCGGCGCAAAGTGCGTCGGGTGATGCTGCCAACTTACTGATTTAGTGTATGATGGTGTTTTTGAGGTGCTCCAGTGGCTTCTGTTTCTATCAGCTGTCCCTCCTGTTCAGCTACTGACGGGGTGGTGCGTAACGGCAAAAGCACCGCCGGACATCAGCGCTAGCGGAGTGTATACTGGCTTACTATGTTGGCACTGATGAGGGTGTCAGTGAAGTGCTTCATGTGGCAGGAGAAAAAAGGCTGCACCGGTGCGTCAGCAGAATATGTGATACAGGATATATTCCGCTTCCTCGCTCACTGACTCGCTACGCTCGGTCGTTCGACTGCGGCGAGCGGAAATGGCTTACGAACGGGGCGGAGATTTCCTGGAAGATGCCAGGAAGATACTTAACAGGGAAGTGAGAGGGCCGCGGCAAAGCCGTTTTTCCATAGGCTCCGCCCCCCTGACAAGCATCACGAAATCTGACGCTCAAATCAGTGGTGGCGAAACCCGACAGGACTATAAAGATACCAGGCGTTTCCCCCTGGCGGCTCCCTCGTGCGCTCTCCTGTTCCTGCCTTTCGGTTTACCGGTGTCATTCCGCTGTTATGGCCGCGTTTGTCTCATTCCACGCCTGACACTCAGTTCCGGGTAGGCAGTTCGCTCCAAGCTGGACTGTATGCACGAACCCCCCGTTCAGTCCGACCGCTGCGCCTTATCCGGTAACTATCGTCTTGAGTCCAACCCGGAAAGACATGCAAAAGCACCACTGGCAGCAGCCACTGGTAATTGATTTAGAGGAGTTAGTCTTGAAGTCATGCGCCGGTTAAGGCTAAACTGAAAGGACAAGTTTTGGTGACTGCGCTCCTCCAAGCCAGTTACCTCGGTTCAAAGAGTTGGTAGCTCAGAGAACCTTCGAAAAACCGCCCTGCAAGGCGGTTTTTTCGTTTTCAGAGCAAGAGATTACGCGCAGACCAAAACGATCTCAAGAAGATCATCTTATTAATCAGATAAAATATTTCTAGATTTCAGTGCAATTTATCTCTTCAAATGTAGCACCTGAAGTCAGCCCCATACGATATAAGTTGTAATTCTCACGGGCGATTTTTATTACGATAATAAAGTCTGTTTTTAATATTATCATGTTAAATGTTTATATTATAAAAAGTCGTTTTTCTGCTTAGGATTTTGTTATTTAAATTAAGCCTGTAATGCCTTGCTTCCATTGCGGATAAATCCTACTTTTTTATTGCCTTCAAATAAATTTAAGGAGTTCGAAATGGCGAGTAGCGAAGACGTTATCAAAGAGTTCATGCGTTTCAAAGTTCGTATGGAAGGTTCCGTTAACGGTCACGAGTTCGAAATCGAAGGTGAAGGTGAAGGTCGTCCGTACGAAGGTACCCAGACCGCTAAACTGAAAGTTACCAAAGGTGGTCCGCTGCCGTTCGCTTGGGACATCCTGTCCCCGCAGTTCCAGTACGGTTCCAAAGCTTACGTTAAACACCCGGCTGACATCCCGGACTACCTGAAACTGTCCTTCCCGGAAGGTTTCAAATGGGAACGTGTTATGAACTTCGAAGACGGTGGTGTTGTTACCGTTACCCAGGACTCCTCCCTGCAAGACGGTGAGTTCATCTACAAAGTTAAACTGCGTGGTACCAACTTCCCGTCCGACGGTCCGGTTATGCAGAAAAAAACCATGGGTTGGGAAGCTTCCACCGAACGTATGTACCCGGAAGACGGTGCTCTGAAAGGTGAAATCAAAATGCGTCTGAAACTGAAAGACGGTGGTCACTACGACGCTGAAGTTAAAACCACCTACATGGCTAAAAAACCGGTTCAGCTGCCGGGTGCTTACAAAACCGACATCAAACTGGACATCACCTCCCACAACGAAGACTACACCATCGTTGAACAGTACGAACGTGCTGAAGGTCGTCACTCCACCGGTGCTTAAGGGATCCAAACTCGAGTAAGGATCTCCAGGCATCAAATAAAACGAAAGGCTCAGTCGAAAGACTGGGCCTTTCGTTTTATCTGTTGTTTGTCGGTGAACGCTCTCTACTAGAGTCACACTGGCTCACCTTCGGGTGGGCCTTTCTGCGTTTATACCTAGGGCGTTCGGCTGCGGCTGGCGCTGGGCCTGTTTCTGGCGCTGGGCGTTTAAGGGCACCAATAACTGCCTTAAAAAAATTACGCCCCGCCCTGCCACTCATCGCAGTACTGTTGTAATTCATTAAGCATTCTGCCGACATGGAAGCCATCACAGACGGCATGATGAACCTGAATCGCCAGCGGCATCAGCACCTTGTCGCCTTGCGTATAATATTTGCCCATGGTGAAAACGGGGGCGAAGAAGTTGTCCATATTGGCCACGTTTAAATCAAAACTGGTGAAACTCACCCAGGGATTGGCTGAGACGAAAAACATATTCTCAATAAACCCTTTAGGGAAATAGGCCAGGTTTTCACCGTAACACGCCACATCTTGCGAATATATGTGTAGAAACTGCCGGAAATCGTCGTGGTATTCACTCCAGAGCGATGAAAACGTTTCAGTTTGCTCATGGAAAACGGTGTAACAAGGGTGAACACTATCCCATATCACCAGCTCACCGTCTTTCATTGCCATACGGAATTCCGGATGAGCATTCATCAGGCGGGCAAGAATGTGAATAAAGGCCGGATAAAACTTGTGCTTATTTTTCTTTACGGTCTTTAAAAAGGCCGTAATA


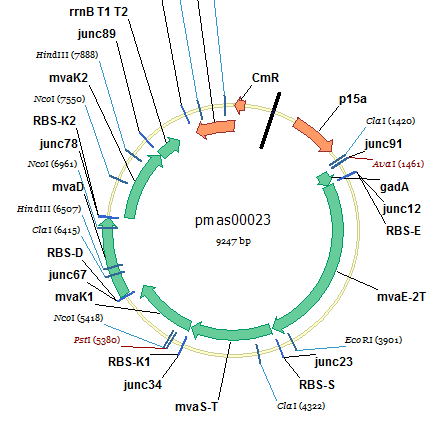


> pACYC184-AL-mva-23

TCCAGCTGAACGGTCTGGTTATAGGTACATTGAGCAACTGACTGAAATGCCTCAAAATGTTCTTTACGATGCCATTGGGATATATCAACGGTGGTATATCCAGTGATTTTTTTCTCCATTTTAGCTTCCTTAGCTCCTGAAAATCTCGATAACTCAAAAAATACGCCCGGTAGTGATCTTATTTCATTATGGTGAAAGTTGGAACCTCTTACGTGCCGATCAACGTCTCATTTTCGCCAAAAGTTGGCCCAGGGCTTCCCGGTATCAACAGGGACACCAGGATTTATTTATTCTGCGAAGTGATCTTCCGTCACAGGTATTTATTCGGCGCAAAGTGCGTCGGGTGATGCTGCCAACTTACTGATTTAGTGTATGATGGTGTTTTTGAGGTGCTCCAGTGGCTTCTGTTTCTATCAGCTGTCCCTCCTGTTCAGCTACTGACGGGGTGGTGCGTAACGGCAAAAGCACCGCCGGACATCAGCGCTAGCGGAGTGTATACTGGCTTACTATGTTGGCACTGATGAGGGTGTCAGTGAAGTGCTTCATGTGGCAGGAGAAAAAAGGCTGCACCGGTGCGTCAGCAGAATATGTGATACAGGATATATTCCGCTTCCTCGCTCACTGACTCGCTACGCTCGGTCGTTCGACTGCGGCGAGCGGAAATGGCTTACGAACGGGGCGGAGATTTCCTGGAAGATGCCAGGAAGATACTTAACAGGGAAGTGAGAGGGCCGCGGCAAAGCCGTTTTTCCATAGGCTCCGCCCCCCTGACAAGCATCACGAAATCTGACGCTCAAATCAGTGGTGGCGAAACCCGACAGGACTATAAAGATACCAGGCGTTTCCCCCTGGCGGCTCCCTCGTGCGCTCTCCTGTTCCTGCCTTTCGGTTTACCGGTGTCATTCCGCTGTTATGGCCGCGTTTGTCTCATTCCACGCCTGACACTCAGTTCCGGGTAGGCAGTTCGCTCCAAGCTGGACTGTATGCACGAACCCCCCGTTCAGTCCGACCGCTGCGCCTTATCCGGTAACTATCGTCTTGAGTCCAACCCGGAAAGACATGCAAAAGCACCACTGGCAGCAGCCACTGGTAATTGATTTAGAGGAGTTAGTCTTGAAGTCATGCGCCGGTTAAGGCTAAACTGAAAGGACAAGTTTTGGTGACTGCGCTCCTCCAAGCCAGTTACCTCGGTTCAAAGAGTTGGTAGCTCAGAGAACCTTCGAAAAACCGCCCTGCAAGGCGGTTTTTTCGTTTTCAGAGCAAGAGATTACGCGCAGACCAAAACGATCTCAAGAAGATCATCTTATTAATCAGATAAAATATTTCTAGATTTCAGTGCAATTTATCTCTTCAAATGTAGCACCTGAAGTCAGCCCCATACGATATAAGTTGTAATTCTCATGTTTGACAGCTTATCATCGATTTAAATTTAATTAAGTGTAGGCTGGAGCTGCTTCGGCTCGGGCGATTTTTATTACGATAATAAAGTCTGTTTTTAATATTATCATGTTAAATGTTTATATTATAAAAAGTCGTTTTTCTGCTTAGGATTTTGTTATTTAAATTAAGCCTGTAATGCCTTGCTTCCATTGCGGATAAATCCTACTTTTTTATTGCCTTCAAATAAATTTACGGTAGGAGGGAGTAAATGAAAACAGTAGTTATTATTGATGCATTACGAACACCAATTGGAAAATATAAAGGCAGCTTAAGTCAAGTAAGTGCCGTAGACTTAGGAACACATGTTACAACACAACTTTTAAAAAGACATTCCACTATTTCTGAAGAAATTGATCAAGTAATCTTTGGAAATGTTTTACAAGCTGGAAATGGCCAAAATCCCGCACGACAAATAGCAATAAACAGCGGTTTATCTCATGAAATTCCCGCAATGACAGTTAATGAGGTCTGCGGATCAGGAATGAAGGCCGTTATTTTGGCGAAACAATTGATTCAATTAGGAGAAGCGGAAGTTTTAATTGCTGGCGGGATTGAGAATATGTCCCAAGCACCTAAATTACAACGATTTAATTACGAAACAGAAAGCTATGATGCGCCTTTTTCTAGTATGATGTACGATGGGTTAACGGATGCCTTTAGTGGTCAAGCAATGGGCTTAACTGCTGAAAATGTGGCCGAAAAGTATCATGTAACTAGAGAAGAGCAAGATCAATTTTCTGTACATTCACAATTAAAAGCAGCTCAAGCACAAGCAGAAGGGATATTCGCTGACGAAATAGCCCCATTAGAAGTATCAGGAACGCTTGTGGAGAAAGATGAAGGGATTCGCCCTAATTCGAGCGTTGAGAAGCTAGGAACGCTTAAAACAGTTTTTAAAGAAGACGGTACTGTAACAGCAGGGAATGCATCAACCATTAATGATGGGGCTTCTGCTTTGATTATTGCTTCACAAGAATATGCCGAAGCACACGGTCTTCCTTATTTAGCTATTATTCGAGACAGTGTGGAAGTCGGTATTGATCCAGCCTATATGGGAATTTCGCCGATTAAAGCCATTCAAAAACTGTTAGCGCGCAATCAACTTACTACGGAAGAAATTGATCTGTATGAAATCAACGAAGCATTTGCAGCAACTTCAATCGTGGTCCAAAGAGAACTGGCTTTACCAGAGGAAAAGGTCAACATTTATGGTGGCGGTATTTCATTAGGTCATGCGATTGGTGCCACAGGTGCTCGTTTATTAACGAGTTTAAGTTATCAATTAAATCAAAAAGAAAAGAAATATGGAGTGGCTTCTTTATGTATCGGCGGTGGCTTAGGACTCGCTATGCTACTAGAGCGTCCTCAGCAAAAAAAAAACAGCCGATTTTATCAAATGAGTCCTGAGGAACGCCTGGCTTCTCTTCTTAATGAAGGCCAGATTTCTGCTGATACAAAAAAAGAATTTGAAAATACGGCTTTATCTTCGCAGATTGCCAATCATATGATTGAAAATCAAATCAGTGAAACAGAAGTGCCGATGGGCGTTGGCTTACATTTAACAGTGGACGAAACTGATTATTTGGTACCAATGGCGACAGAAGAGCCCTCAGTGATTGCGGCTTTGAGTAATGGTGCAAAAATAGCACAAGGATTTAAAACAGTGAATCAACAACGTTTAATGCGTGGACAAATCGTTTTTTACGATGTTGCAGACGCCGAGTCATTGATTGATGAACTACAAGTAAGAGAAACGGAAATTTTTCAACAAGCAGAGTTAAGTTATCCATCTATCGTTAAACGCGGCGGCGGCTTAAGAGATTTGCAATATCGTGCTTTTGATGAATCATTTGTATCTGTCGACTTTTTAGTAGATGTTAAGGATGCAATGGGGGCAAATATCGTTAACGCTATGTTGGAAGGTGTGGCCGAGTTGTTCCGTGAATGGTTTGCGGAGCAAAAGATTTTATTCAGTATTTTAAGTAATTATGCCACGGAGTCGGTTGTTACGATGAAAACGGCTATTCCAGTTTCACGTTTAAGTAAGGGGAGCAATGGCCGGGAAATTGCTGAAAAAATTGTTTTAGCTTCACGCTATGCTTCATTAGATCCTTATCGGGCAGTCACGCATAACAAAGGGATCATGAATGGCATTGAAGCTGTCGTTTTAGCTACAGGAAATGATACACGCGCTGTTAGCGCTTCTTGTCATGCTTTTGCGGTGAAGGAAGGTCGCTACCAAGGTTTGACTAGTTGGACGCTGGATGGCGAACAACTAATTGGTGAAATTTCAGTTCCGCTTGCGTTAGCCACGGTTGGCGGTGCCACAAAAGTCTTACCTAAATCTCAAGCAGCTGCTGATTTGTTAGCAGTGACGGATGCAAAAGAACTAAGTCGAGTAGTAGCGGCTGTTGGTTTGGCACAAAATTTAGCGGCGTTACGGGCCTTAGTCTCTGAAGGAATTCAAAAAGGACACATGGCTCTACAAGCACGTTCTTTAGCGATGACGGTCGGAGCTACTGGTAAAGAAGTTGAGGCAGTCGCTCAACAATTAAAACGTCAAAAAACGATGAACCAAGACCGAGCCTTGGCTATTTTAAATGATTTAAGAAAACAATAAGTGCAGGAGGTGGGTGATGACAATTGGGATTGATAAAATTAGTTTTTTTGTGCCCCCTTATTATATTGATATGACGGCACTGGCTGAAGCCAGAAATGTAGACCCTGGAAAATTTCATATTGGTATTGGGCAAGACCAAATGGCGGTGAACCCAATCAGCCAAGATATTGTGACATTTGCAGCCAATGCCGCAGAAGCGATCTTGACCAAAGAAGATAAAGAGGCCATTGATATGGTGATTGTCGGGACTGAGTCCAGTATCGATGAGTCAAAAGCGGCCGCAGTTGTCTTACATCGTTTAATGGGGATTCAACCTTTCGCTCGCTCTTTCGAAATCAAGGAAGGTTGTTACGGAGCAACAGCAGGCTTACAGTTAGCTAAGAATCACGTAGCCTTACATCCAGATAAAAAAGTCTTGGTTGTAGCAGCAGATATTGCAAAATATGGATTAAATTCTGGCGGTGAGCCTACACAAGGAGCTGGGGCGGTTGCAATGTTAGTTGCTAGTGAACCGCGCATCTTGGCTTTAAAAGAGGATAATGTGATGCTGACGCAAGATATCTATGACTTTTGGCGTCCAACAGGCCATCCGTATCCTATGGTCGATGGTCCTTTGTCAAACGAAACCTACATCCAATCTTTTGCCCAAGTCTGGGATGAACATAAAAAAAGAACCGGTCTTGATTTTGCAGATTATGATGCTTTAGCGTTCCATATTCCTTACACAAAAATGGGCAAAAAAGCCTTATTAGCAAAAATCTCCGACCAAACTGAAGCAGAACAGGAACGAATTTTAGCCCGTTATGAAGAAAGCATCATCTATAGTCGTCGCGTAGGAAACTTGTATACGGGTTCACTTTATCTGGGACTCATTTCCCTTTTAGAAAATGCAACGACTTTAACCGCAGGCAATCAAATTGGGTTATTCAGTTATGGTTCTGGTGCTGTCGCTGAATTTTTCACTGGTGAATTAGTAGCTGGTTATCAAAATCATTTACAAAAAGAAACTCATTTAGCACTGCTAGATAATCGGACAGAACTTTCTATCGCTGAATATGAAGCCATGTTTGCAGAAACTTTAGACACAGATATTGATCAAACGTTAGAAGATGAATTAAAATATAGTATTTCTGCTATTAATAATACCGTTCGCTCTTATCGAAACTAAAGCGAGGAGAGGGTCCATGACAAAAAAAGTTGGTGTCGGTCAGGCACATAGTAAGATAATTTTAATAGGGGAACATGCGGTCGTTTACGGTTATCCTGCCATTTCCCTGCCTCTTTTGGAGGTGGAGGTGACCTGTAAGGTAGTTTCTGCAGAGAGTCCTTGGCGCCTTTATGAGGAGGATACCTTGTCCATGGCGGTTTATGCCTCACTGGAGTATTTGGATATCACAGAAGCCTGCGTTCGTTGTGAGATTGACTCGGCTATCCCTGAGAAACGGGGGATGGGTTCGTCAGCGGCTATCAGCATAGCGGCCATTCGTGCGGTATTTGACTACTATCAGGCTGATCTGCCTCATGATGTACTAGAAATCTTGGTCAATCGAGCTGAGATGATTGCCCATATGAATCCTAGTGGTTTGGATGCTAAGACCTGTCTCAGTGACCAACCTATTCGCTTTATCAAGAACGTAGGATTTACAGAACTTGAGATGGATTTATCCGCCTATTTGGTGATTGCCGATACGGGTGTTTATGGTCATACTCGTGAAGCCATCCAAGTGGTTCAAAATAAGGGCAAGGATGCCCTACCGTTTTTGCATGCCTTGGGAGAATTAACCCAGCAAGCAGAAGTTGCGATTTCACAAAAATATGCTGAAGGACTGGGACTAATCTTCAGTCAAGCTCATTTACATCTAAAAGAAATTGGAGTCAGTAGCCCTGAGGCAGACTTTTTGGTTGAAACGGCTCTTAGCTATGGTGCTCTGGGTGCCAAGATGAGCGGTGGTGGGCTAGGAGGTTGTATCATAGCCTTGGTAACCAATTTGACGCACGCACAAGAACTAGCAGAAAGATTAGAAGAGAAAGGAGCTGTTCAGACATGGATAGAGAGCCTGTAAGGACAGGAGGTGCCATATGGATAGAGAGCCTGTAACAGTACGTTCCTACGCAAATATTGCTATTATCAAATATTGGGGAAAGAAAAAAGAAAAAGAGATGGTGCCTGCTACTAGCAGTATTTCTCTAACTTTGGAAAATATGTATACAGAGACGACCTTGTCGCCTTTACCAGCCAATGTAACAGCTGACGAATTTTACATCAATGGTCAGCTACAAAATGAGGTCGAGCATGCCAAGATGAGTAAGATTATTGACCGTTATCGTCCAGCTGGTGAGGGCTTTGTCCGTATCGATACTCAAAACAATATGCCTACGGCAGCGGGCCTGTCCTCAAGTTCTAGTGGTTTGTCCGCCCTGGTCAAGGCTTGTAATGCTTATTTCAAGCTTGGATTGGATAGAAGTCAGTTGGCACAGGAAGCCAAATTTGCCTCAGGCTCTTCTTCTCGGAGTTTTTATGGACCACTAGGAGCCTGGGATAAGGATAGTGGAGAAATTTACCCTGTAGAGACAGACTTGAAACTAGCTATGATTATGTTGGTGCTAGAGGACAAGAAAAAACCAATCTCTAGCCGTGACGGGATGAAACTTTGTGTGGAAACCTCGACGACTTTTGACGACTGGGTTCGTCAGTCTGAGAAGGACTATCAGGATATGCTGATTTATCTCAAGGAAAATGATTTTGCCAAGATTGGAGAATTAACGGAGAAAAATGCTCTGGCTATGCATGCTACGACAAAGACTGCTAGTCCAGCCTTTTCTTATCTGACGGATGCCTCTTATGAGGCTATGGCCTTTGTTCGCCAGCTTCGTGAGAAAGGAGAGGCCTGCTACTTTACCATGGATGCTGGTCCCAATGTTAAGGTCTTCTGTCAGGAGAAAGACTTGGAGCATTTGTCAGAAATTTTCGGTCAGCGTTATCGCTTGATTGTGTCAAAAACAAAGGATTTGAGTCAAGATGATTGCTGTTAATCCGAGGAGGGATGCGATGATTGCTGTTAAAACTTGCGGAAAACTCTATTGGGCAGGTGAATATGCTATTTTAGAGCCAGGGCAGTTAGCTTTGATAAAGGATATTCCCATCTATATGAGGGCTGAGATTGCTTTTTCTGACAGCTACCGTATCTATTCAGATATGTTTGATTTCGCAGTGGACTTAAGGCCCAATCCTGACTACAGCTTGATTCAAGAAACGATTGCTTTGATGGGAGACTTCCTCGCTGTTCGCGGTCAGAATTTAAGACCTTTTTCCCTAAAAATCTGTGGCAAAATGGAACGAGAAGGGAAAAAGTTTGGTCTAGGTTCTAGTGGCAGCGTCGTTGTCTTGGTTGTCAAGGCTTTACTGGCTCTCTATAATCTTTCGGTTGATCAGAATCTCTTGTTCAAGCTGACTAGCGCTGTCTTGCTCAAGCGAGGAGACAATGGTTCCATGGGCGACCTTGCCTGTATTGTGGCAGAGGATTTGGTTCTTTACCAGTCATTTGATCGCCAGAAGGCGGCTGCTTGGTTAGAAGAAGAAAACTTGGCGACAGTTCTGGAGCGTGATTGGGGATTTTTTATCTCACAAGTGAAACCAACTTTAGAATGTGATTTCTTAGTGGGATGGACCAAGGAAGTGGCTGTATCGAGTCACATGGTCCAGCAAATCAAGCAAAATATCAATCAAAATTTTTTAAGTTCCTCAAAAGAAACGGTGGTTTCTTTGGTCGAAGCCTTGGAGCAGGGGAAAGCCGAAAAAGTTATCGAGCAAGTAGAAGTAGCCAGCAAGCTTTTAGAAGGCTTGAGTACAGATATTTACACGCCTTTGCTTAGACAGTTGAAAGAAGCCAGTCAAGATTTGCAGGCCGTTGCCAAGAGTAGTGGTGCTGGTGGTGGTGACTGTGGCATCGCCCTGAGTTTTGATGCGCAATCTTCTCGAAACACTTTAAAAAATCGTTGGGCCGATCTGGGGATTGAGCTCTTATATCAAGAAAGGATAGGACATGACGACAAATCGTAACCAGGAATTTGCCTGGCGGCAGTAGCGCGGTGGTCCCACCTGACCCCATGCCGAACTCAGAAGTGAAACGCCGTAGCGCCGATGGTAGTGTGGGGTCTCCCCATGCGAGAGTAGGGAACTGCCAGGCATCAAATAAAACGAAAGGCTCAGTCGAAAGACTGGGCCTTTCGTTTTATCTGTTGTTTGTCGGTGAACGCTCTCCTGAGTAGGACAAATCCGCCGGGAGCGGATTTGAACGTTGCGAAGCAACGGCCCGGAGGGTGGCGGGCAGGACGCCCGCCATAAACTGCCAGGCATCAAATTAAGCAGAAGGCCATCCTGACGGATGGCCTTTTTGCGTTTCTATTTAAATGGGAGAGCCTGAGCAAACTGGCCTCAGGCATTTGAGAAGCACACGGTCACACTGCTTCCGGTAGTCAATAAACCGGTAAACCAGCAATAGACATAAGCGGCTATTTAACGACCCTGCCCTGAACCGACGACCGGGTCGAATTTGCTTTCGAATTTCTGCCATTCATCCGCTTATTATCACTTATTCAGGCGTAGCACCAGGCGTTTAAGGGCACCAATAACTGCCTTAAAAAAATTACGCCCCGCCCTGCCACTCATCGCAGTACTGTTGTAATTCATTAAGCATTCTGCCGACATGGAAGCCATCACAGACGGCATGATGAACCTGAATCGCCAGCGGCATCAGCACCTTGTCGCCTTGCGTATAATATTTGCCCATGGTGAAAACGGGGGCGAAGAAGTTGTCCATATTGGCCACGTTTAAATCAAAACTGGTGAAACTCACCCAGGGATTGGCTGAGACGAAAAACATATTCTCAATAAACCCTTTAGGGAAATAGGCCAGGTTTTCACCGTAACACGCCACATCTTGCGAATATATGTGTAGAAACTGCCGGAAATCGTCGTGGTATTCACTCCAGAGCGATGAAAACGTTTCAGTTTGCTCATGGAAAACGGTGTAACAAGGGTGAACACTATCCCATATCACCAGCTCACCGTCTTTCATTGCCATACGGAATTCCGGATGAGCATTCATCAGGCGGGCAAGAATGTGAATAAAGGCCGGATAAAACTTGTGCTTATTTTTCTTTACGGTCTTTAAAAAGGCCGTAATA
